# Circulating VEGF-A, TNF-α, CCL2, IL-6, and IFN-γ as biomarkers of cancer in cancer-associated anti-TIF1-γ antibody-positive dermatomyositis

**DOI:** 10.1007/s10067-022-06425-3

**Published:** 2022-11-11

**Authors:** Xiaomeng Li, Yuan Huang, Yongmei Liu, Songxin Yan, Liubing Li, Linlin Cheng, Haolong Li, Haoting Zhan, Fengchun Zhang, Yongzhe Li

**Affiliations:** 1 Department of Clinical Laboratory, State Key Laboratory of Complex Severe and Rare Diseases, Peking Union Medical College Hospital, Chinese Academy of Medical Science and Peking Union Medical College, Beijing, China; 2 Department of Medical Research Center, Peking Union Medical College Hospital, Chinese Academy of Medical Science and Peking Union Medical College, Beijing, China; 3 Department of Medicine, Peking Union Medical College Hospital, Chinese Academy of Medical Sciences and Peking Union Medical College, Beijing, China

**Keywords:** Anti-TIF1-γ antibody, Biomarker, Cancer, Cytokine, Dermatomyositis

## Abstract

**Objectives:**

The objective of the current study was to detect plasma profiles of inflammatory cytokines for determining potential biomarkers indicating cancer presence among the anti-TIF1-γ antibody-positive dermatomyositis (DM) patients.

**Methods:**

Twenty-seven cancer-associated anti-TIF1-γ antibody-positive DM (Cancer TIF1-γ-DM) patients were compared with 20 anti-TIF1-γ antibody-positive DM patients without cancer (Non-cancer TIF1-γ-DM) and 10 healthy controls (HC). The plasma levels of 17 cytokines were determined using the Luminex 200 system. The ability of plasma VEGF-A, TNF-α, CCL2, IL-6, and IFN-γ levels to distinguish the presence of cancer was evaluated through the area under the curve (AUC) analysis. Potential protein interactions of TIF1-γ and the five cytokines were analyzed using the STRING database.

**Results:**

VEGF-A, TNF-α, CCL2, IL-6, and IFN-γ plasma levels were significantly higher in the Cancer TIF1-γ-DM group, especially those without any anticancer treatment, than those in the non-cancer TIF1-γ-DM and HC groups. Meanwhile, anti-TIF1-γ antibody and the five cytokines could distinguish cancer presence in anti-TIF1-γ antibody-positive DM patients. The STRING network indicated that TIF1-γ potentially interacted with the cytokines. Positive correlations of VEGF-A among CCL2, IL-6, and IFN-γ and between IFN-γ and IL-6 were observed in Cancer TIF1-γ-DM patients. VEGF-A, TNF-α, CCL2, and IL-6 were positively associated with muscle-associated enzymes among the Cancer TIF1-γ-DM patients.

**Conclusion:**

The present study identified VEGF-A, TNF-α, CCL2, IL-6, and IFN-γ as significant potential biomarkers indicating the presence of cancer and demonstrated a more detailed cytokine profile during diagnosis. These biomarkers could provide better screening strategies and insight into the Cancer TIF1-γ-DM pathogenesis.

**Table:** 

**Key Points** *• VEGF-A, TNF-α, CCL2, IL-6, and IFN-γ are potential biomarkers of cancer in cancer-associated anti-TIF1-γ antibody-positive dermatomyositis.*

**Graphical abstract:**

**Figure:**
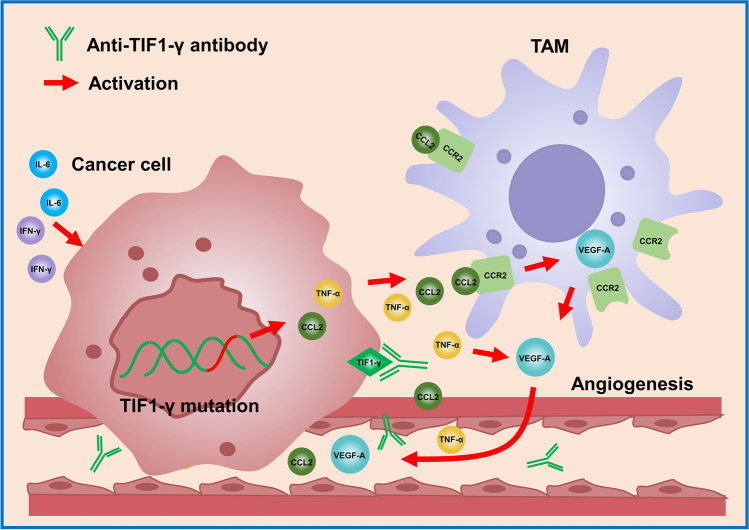
Potential pathogenic molecular mechanism of the cancer-associated anti-TIF1-γ antibody-positive dermatomyositis

**Supplementary Information:**

The online version contains supplementary material available at 10.1007/s10067-022-06425-3.

## Introduction

Dermatomyositis (DM) is a more frequent idiopathic inflammatory myopathy (IIM) with incidence rates of 11–660 per 1,000,000 person-years and prevalence rates of 2.9–34 per 100,000 person-years. It is predominantly characterized by distinct skin lesions, symmetric proximal muscle weakness, and muscle inflammation [1, 2]. Cancer and interstitial lung disease (ILD) are the leading causes of mortality among DM patients. The epidemiological evidence reveals that the risk of cancer-associated myositis (CAM) is significantly higher in DM (standardized incidence ratio 4.66) than in other IIM subtypes [3]. DM is strongly associated with various cancers [4]. However, the routine clinical use of tumor markers, including carcinoembryonic antigen (CEA), CA15-3, and CA19-9, were not elevated significantly in CAM, except CA125 [5]. Moreover, myositis-specific autoantibodies (MSAs), including anti-transcriptional intermediary factor 1-gamma (TIF1-γ), anti-nuclear matrix protein-2 (NXP-2), anti-small ubiquitin-like modifier activating enzyme (SAE) and anti-3-hydroxy-3-methylglutaryl-coenzyme A reductase (HMGCR) autoantibodies, have been associated with an increased risk of cancer in IIM. Numerous studies have shown that DM-specific anti-TIF1-γ autoantibodies (Odds Ratio 27.26) have the highest risk of cancer development [6]. The frequency of anti-TIF1-γ antibody is detected in 30–40% juvenile and 20% adult DM, respectively. However, the strong correlation with cancer is only present among adult DM patients [7]. In a Chinese cohort, the frequency of anti-TIF1-γ antibody was detected among IIM patients, with 61.8% having cancer and 10.4% without cancer [8]. It indicated that not all the anti-TIF1-γ antibody-positive DM patients develop cancer. The reason behind some anti-TIF1-γ antibody-positive adult DM patients with no detectable cancer remains unclear.

DM is predominantly mediated through humoral immunity involving B cells, CD4^+^ T cells, macrophages, muscle infiltration, antibodies, and complement-mediated injury of capillaries accompanied by inflammatory cytokine dysregulations [9, 10]. Positive correlations between disease severity and serum cytokine levels, including C-X-C motif chemokine ligand 10 (CXCL10), C-C motif chemokine ligand 2 (CCL2), Galectin-9, interleukin-18 (IL-18), tumor necrosis factor-α (TNF-α), and TNF receptor 1 (TNFR1) were observed within anti-TIF1-γ antibody-positive DM patients [11]. Autoimmune diseases and cancer have a robust inflammatory background mediated by inflammatory cytokines despite displaying fundamentally different pathological conditions [12]. Some similarities between autoimmunity and cancer have been observed, such as the activation of phagocytes and angiogenesis [13]. Currently, CAM is considered a paraneoplastic myositis syndrome (PMS). The exact underlying pathogenic mechanism of CAM is unknown. However, it is widely suggested that the loss of heterozygosity (LOH) or mutations in TIF1-γ genes inside tumors induce cross-reactivity against wild-type TIF1-γ antigens in the muscle and skin and subsequently leads to cancer-associated anti-TIF1-γ antibody-positive DM development [14, 15]. A recent study revealed that some serum inflammatory cytokines, including the B-cell activating factor of the TNF superfamily (BAFF), soluble TNF receptor 1 (sTNF-R1), and sTNF-R2 could be cancer predictors among anti-TIF1-γ antibody-positive DM patients [16]. Few studies have compared plasma cytokine levels in cancer-associated anti-TIF1-γ antibody-positive DM patients and those without cancer. It is unclear whether plasma inflammatory cytokines serve as biomarkers in diagnosing and surveilling cancer in anti-TIF1-γ antibody-positive DM patients.

Therefore, this study aimed to detect the plasma levels of inflammatory cytokine profiles to identify potential biomarkers reflecting the presence of cancer in anti-TIF1-γ antibody-positive DM patients. Moreover, we attempted to develop targeted cancer screening guidelines and offer insight into the underlying cancer-associated anti-TIF1-γ antibody-positive DM pathogenesis. In the present study, 17 inflammatory cytokines (vascular endothelial growth factor-A, VEGF-A; TNF-α; TNF-β; CCL2; CCL3; IL-1β; IL-2; IL-4; IL-5; IL-6; IL-10; IL-12p70; IL-17A; interferon-α2, IFN-α2; IFN-γ; CXCL10; and soluble CD40 ligand, sCD40L) were evaluated in cancer-associated anti-TIF1-γ antibody-positive DM (Cancer TIF1-γ-DM) patients, anti-TIF1-γ antibody-positive DM patients without cancer (Non-cancer TIF1-γ-DM) and healthy controls (HC). Moreover, for further analysis, the above indicators were compared in untreated and treated Cancer TIF1-γ-DM patients. Significantly higher levels of plasma VEGF-A, TNF-α, CCL2, IL-6, and IFN-γ were observed among Cancer TIF1-γ-DM patients, especially among untreated Cancer TIF1-γ-DM patients. These five circulating biomarkers could reflect the cancer presence in anti-TIF1-γ antibody-positive DM patients.

## Methods

### Study subjects

In this retrospective observational study, consecutive patients were enrolled at the Peking Union Medical College Hospital from July 2013 to December 2021. Patients are eligible for inclusion if they are adults, diagnosed DM based on Bohan and Peter’s criteria and EULAR/ACR criteria [17–19], with an ELISA confirmed a positive expression of anti-TIF1-γ antibody, and cancer-associated anti-TIF1-γ antibody-positive DM (Cancer TIF1-γ-DM) belonged to CAM defined as those patients with cancer occurrence within 3 years (before or after) of DM onset [20] or anti-TIF1-γ antibody-positive DM without cancer (Non-cancer TIF1-γ-DM) including patients with negative cancer detection performed within 3 years using whole-body PET/CT or conventional cancer screening [mammography and gynecological ultrasound exam among women, whole (chest/abdomen/pelvis) computed tomography, gastrointestinal endoscopy, and colonoscopy] [16, 21]. Patients who suffered from overlapping rheumatic disease were excluded. Healthy controls without any known inflammatory diseases, recent infections, or relevant medical history were included to evaluate the cytokine levels.

Clinical data were abstracted from medical records, including patient age, gender, physical examination findings, laboratory data, and treatment history. Plasma (EDTA anticoagulant) samples were collected and stored at −80 °C until further analysis.

### Measurement of anti-TIF1-γ antibody

Plasma anti-TIF1-γ antibody levels within anti-TIF1-γ antibody-positive adult DM patients were detected using an ELISA kit (Medical and Biological Laboratories, Nagoya, Japan) based on the manufacturer’s instructions. Unit values ≥ 32 U/mL were defined as positive [22]. The expression of anti-TIF1-γ antibody was negative measured by ELISA and immunoprecipitation (IP) in the serum of healthy controls [23]. Therefore, this study did not detect the anti-TIF1-γ antibody levels in the HC group.

### Measurement of plasma cytokines

The plasma levels of 17 cytokines, including VEGF-A, TNF-α, TNF-β, CCL2, CCL3, IL-1β, IL-2, IL-4, IL-5, IL-6, IL-10, IL-12p70, IL-17A, IFN-α2, IFN-γ, CXCL10, and sCD40L, were measured using the Luminex 200 system (Luminex Corporation, Austin, TX, USA) through a MILLIPLEX MAP Human Cytokine/Chemokine/Growth Factor Panel A-Immunology Multiplex Assay (MilliporeSigma, Billerica, MA, USA) kit based on the manufacturer’s instructions. The concentrations of cytokines below the detection limit were considered half of the detection limit [24].

### Statistical analysis

Based on the data distribution, differences between the two groups of continuous variables were compared using the unpaired, two-tailed *t*-test or Mann-Whitney *U* test. Differences among the multiple groups of continuous variables were compared through the One-way analysis of variance (ANOVA) followed by Tukey’s multiple comparisons test (normally distributed data) or Kruskal-Wallis test followed by Dunn’s multiple comparisons test (nonparametric data). For the 17 tested cytokines, before and after Bonferroni correction was adopted with significance at *P* < 0.05 and *P* < 0.0029 (0.05 divided by 17), respectively. Categorical variables were compared using Fisher’s exact test. Hierarchical cluster analysis was performed through Euclidian distance and the Ward agglomerative method using Hiplot (https://hiplot.org). The area under the curve (AUC) was determined using the receiver operating characteristic (ROC) curve analysis. The STRING (https://string-db.org/, version 11.5) database was used for online protein-protein interaction analysis. Spearman’s rank correlation test was utilized for correlation analysis. The sensitivity, specificity, positive and negative predictive values of PET/CT or usual screening, anti-TIF1-γ antibody, VEGF-A, TNF-α, CCL2, IL-6, and IFN-γ in the Cancer TIF1-γ-DM and Non-cancer TIF1-γ-DM diagnosis were separately calculated based on the gold standard of clinical diagnosis. The statistical significance was indicated by two-sided *P* values < 0.05. The statistical analyses were performed using the GraphPad Prism 9 (GraphPad Software, La Jolla, CA, USA) and the SPSS Statistics 26 (IBM, Chicago, IL, USA) software.

## Results

### Subject characteristics

Forty-seven anti-TIF1-γ antibody-positive adult DM patients and ten healthy controls (HC group) were enrolled in this study. Of these 47 patients, 27 had complicated cancer (classified as Cancer TIF1-γ-DM group), and 20 were without cancer (classified as Non-cancer TIF1-γ-DM group). Forty-six of 47 patients had undergone treatment intervention regarding DM, including glucocorticoids, immunosuppressants, biological agents, or traditional Chinese medicine, while one did not receive any treatment. For cancer treatment, 19 of the 27 Cancer TIF1-γ-DM patients had undergone surgical intervention, radiotherapy, or chemotherapy (classified as Treated Cancer TIF1-γ-DM group). Eight of the 27 Cancer TIF1-γ-DM patients (classified as Untreated Cancer TIF1-γ-DM group) did not receive anticancer treatment before plasma sample collection. Table 1 indicates that when plasma was collected, the Cancer TIF1-γ-DM patients were significantly older than Non-cancer TIF1-γ-DM patients, even when diagnosed with DM. A higher proportion of males and higher erythrocyte sedimentation rate (ESR) levels belonged to the Cancer TIF1-γ-DM group than the Non-cancer TIF1-γ-DM group. The sex distribution in both the groups was different because all the patients were female in the Non-cancer TIF1-γ-DM group. The levels of muscle enzyme lactate dehydrogenase (LDH) were significantly higher in the Cancer TIF1-γ-DM and Non-cancer TIF1-γ-DM groups than in the HC group. Additionally, the Cancer TIF1-γ-DM group patients had significantly more elevated aspartate aminotransferase (AST) levels than the HC group.

**Table 1:** Demographic and clinical characteristics of the study groups

Characteristics at the time of plasma collection	Cancer TIF1-γ-DM [*n* = 27]	Non-cancer TIF1-γ-DM [*n* = 20]	HC [*n* = 10]	*P* value		
				Cancer TIF1-γ-DM vs. Non-cancer TIF1-γ-DM	Cancer TIF1-γ-DM vs. HC	Non-cancer TIF1-γ-DM vs. HC
Sex, *n* (%)				**0.0025**^a^	> 0.9999^a^	**0.0296**^a^
Male	10 (37)	0 (0)	3 (30)	-	-	-
Female	17 (63)	20 (100)	7 (70)	-	-	-
Age, mean ± SD years						
Age	60.33 ± 10.13	49.70 ± 12.53	55.70 ± 5.658	**0.0032**^b^	0.4624^b^	0.3100^b^
Age at DM diagnosis	59.67 ± 9.985	47.70 ± 13.84	-	**0.0012**^c^	-	-
Age at cancer diagnosis	59.29 ± 10.13*	-	-	-	-	-
DM duration						
median (Q1, Q3), month	2.000 (0, 12.00)	3.000 (0, 14.75)	-	0.5843^d^	-	-
Clinical features, *n* (%)						
Interstitial lung disease	1 (4)	2 (10)	-	0.5671^a^	-	-
Heliotrope rash	11 (41)	10(50)	-	0.5661^a^	-	-
Gottron's papules/sign	10 (37)	12 (60)	-	0.1476^a^	-	-
V sign	7 (26)	10 (50)	-	0.1273^a^	-	-
Shawl sign	4 (15)	7 (35)	-	0.1645^a^	-	-
Periungual erythema	7 (26)	5 (25)	-	> 0.9999^a^	-	-
Muscle weakness	23 (85)	12 (60)	-	0.0890^a^	-	-
Dysphagia	9 (33)	3 (15)	-	0.1913^a^	-	-
Treatment, *n* (%)						
Glucocorticoids	23 (85)	15 (75)	-	0.4653^a^	-	-
Immunosuppressants	11 (41)	11 (55)	-	0.3860^a^	-	-
Biological agent	0 (0)	3 (15)	-	0.0703^a^	-	-
Traditional Chinese medicine	0 (0)	1 (5)	-	0.4255^a^	-	-
No therapy	1 (4)	0 (0)	-	> 0.9999^a^	-	-
Laboratory data, median (Q1, Q3)						
CK, U/L	127.5 (47.25, 392.5) [*n* = 18]	67.00 (43.50, 132.0) [*n* = 20]	-	0.3135^d^	-	-
LDH, U/L	320.0 (270.5, 368.0) [*n* = 21]	270.0 (191.0, 344.0) [*n* = 19]	172.0 (137.5, 197.3) [*n* = 8]	0.1951^e^	**< 0.0001**^e^	**0.0176**^e^
ALT, U/L	22.50 (17.50, 42.00) [*n* = 26]	26.00 (16.00, 36.00) [*n* = 19]	16.50 (13.00, 21.25) [n = 10]	**>** 0.9999^e^	0.1455^e^	0.1733^e^
AST, U/L	35.00 (24.00, 57.50) [*n* = 21]	23.50 (14.50, 37.75) [*n* = 18]	19.50 (18.00, 21.50) [*n* = 8]	0.1458^e^	**0.0355**^e^	0.9964^e^
ESR, mm/h	20.50 (12.50, 28.50) [*n* = 14]	8.000 (6.750, 13.75) [*n* = 14]	-	**0.0036**^d^	-	-
CRP, mg/L	1.320 (0.7150, 12.18) [*n* = 13]	2.995 (0.6600, 4.628) [*n* = 16]	-	0.8799^d^	-	-

*DM*, dermatomyositis; *TIF1-γ*, transcriptional intermediary factor 1-γ; *HC*, healthy controls; *CK*, creatine kinase; *LDH*, lactate dehydrogenase; *ALT*, alanine aminotransferase; *AST*, aspartate aminotransferase; *ESR*, erythrocyte sedimentation rate; *CRP*, C-reactive protein.

*One patient had double cancers. The patient was diagnosed with thyroid cancer at the age of 56, followed by breast cancer at the age of 57.

^a^Fisher’s exact test.

^b^One-way analysis of variance followed by Tukey’s multiple comparisons test.

^c^Unpaired *t* test.

^d^Mann-Whitney *U* test.

^e^Kruskal-Wallis test followed by Dunn’s multiple comparisons test.

*P* < 0.05 was considered statistically significant in bold text.

**Fig. 1:**
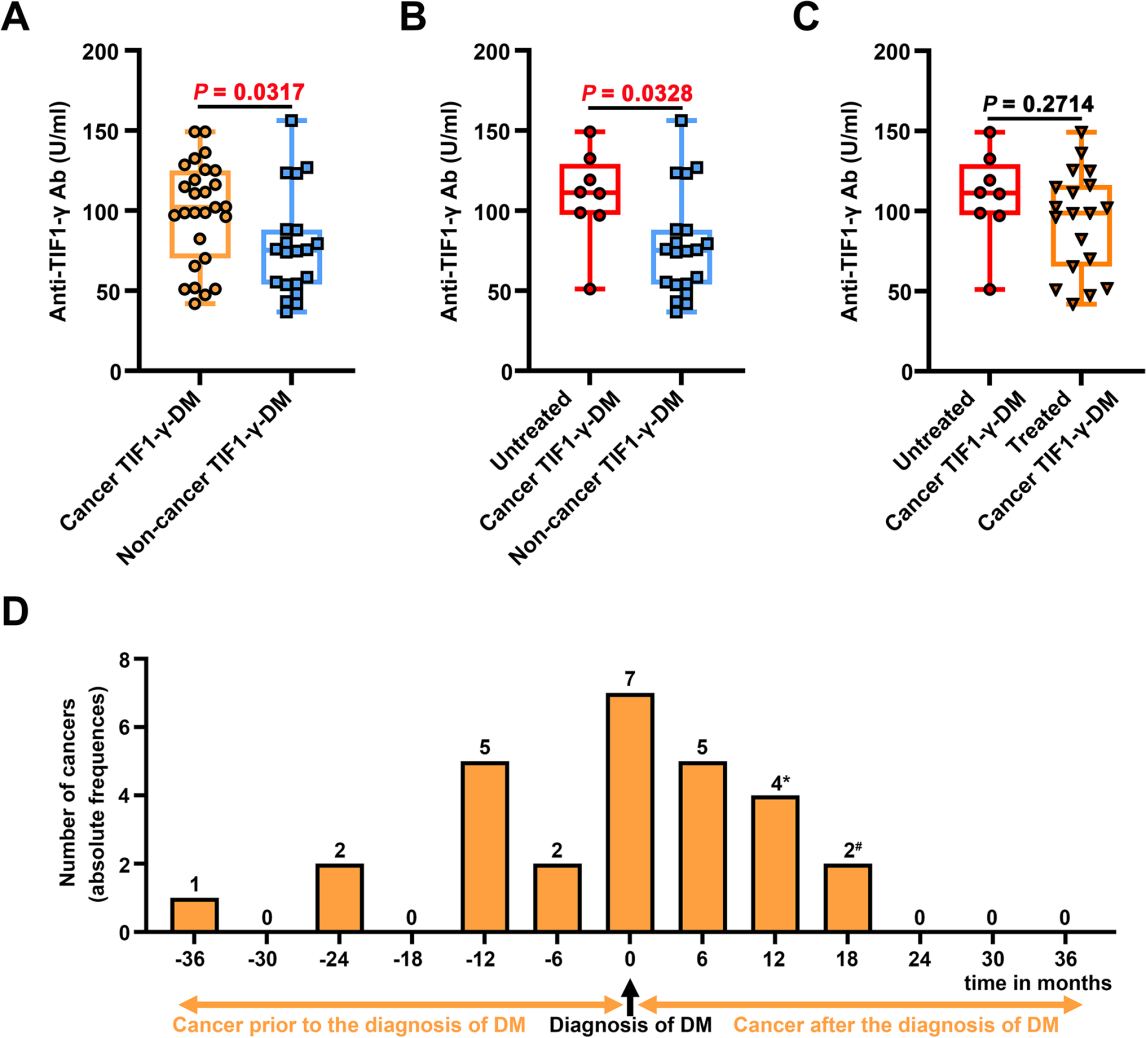
Anti-TIF1-γ antibody plasma levels and the temporal relationship between cancer onset and DM. (**A**) Comparison of the anti-TIF1-γ antibody between the Cancer TIF1-γ-DM and the Non-cancer TIF1-γ-DM groups. (**B**) Comparison of anti-the TIF1-γ antibody between the Untreated Cancer TIF1-γ-DM and the Non-cancer TIF1-γ-DM groups. (**C**) Comparison of the anti-TIF1-γ antibody between the Untreated and Treated in Cancer TIF1-γ-DM patients. (**D**) Temporal relationship between the diagnosis of cancer and DM. * # One patient had double cancer. The patient was diagnosed with* thyroid cancer in the eighth month, # followed by breast cancer in the 18^th^ month after having dermatomyositis. *P* values were determined through the Mann-Whitney *U* test or the unpaired *t-*test. All the data are displayed in boxplots depicting the median with an interquartile range. *P* < 0.05 was considered statistically significant in bold red text

In this study, significantly higher levels of plasma anti-TIF1-γ antibodies were observed among the Cancer TIF1-γ-DM patients than the Non-cancer TIF1-γ-DM patients (Figure 1A). In the Untreated Cancer TIF1-γ-DM group, plasma anti-TIF1-γ antibody levels were significantly higher than in the Non-cancer TIF1-γ-DM group (Figure 1B). Anti-TIF1-γ antibody levels were slightly lower in the Treated than in the Untreated Cancer TIF1-γ-DM patients, though without any statistical difference (Figure 1C).

Twenty-eight cancers were documented among the 27 Cancer TIF1-γ-DM patients (Figure 1D). In particular, most cancers (82%) were diagnosed simultaneously or within the first year (before or after) of DM diagnosis. Overall, lung cancer (32%) and breast cancer (29%) were the most common among Cancer TIF1-γ-DM patients (Table 2). Breast cancer was the most frequent type in female patients, followed by ovarian cancer. In contrast, lung cancer was the most frequent cancer among male patients.

**Table 2:** Types of cancers in Cancer TIF1-γ-DM group (*n* = 27)

Types of cancers	Number		Total, *n* (%)	
	Male	Female		
Breast cancer	-	8	8^*^	(29)
Ovarian cancer	-	4	4	(14)
Fallopian tube cancer	-	1	1	(4)
Lung cancer	6	3	9	(32)
Liver cancer	1	-	1	(4)
Thyroid cancer	-	1	1^*^	(4)
Nasopharynx cancer	-	1	1	(4)
Prostate cancer	1	-	1	(4)
Tonsil cancer	1	-	1	(4)
Neck cancer	1	-	1	(4)
Total	10	18	28	(100)

^*^One patient had double cancers, breast cancer and thyroid cancer, respectively

### Upregulated levels of plasma VEGF-A, TNF-α, CCL2, IL-6, and IFN-γ in Cancer TIF1-γ-DM

**Fig. 2:**
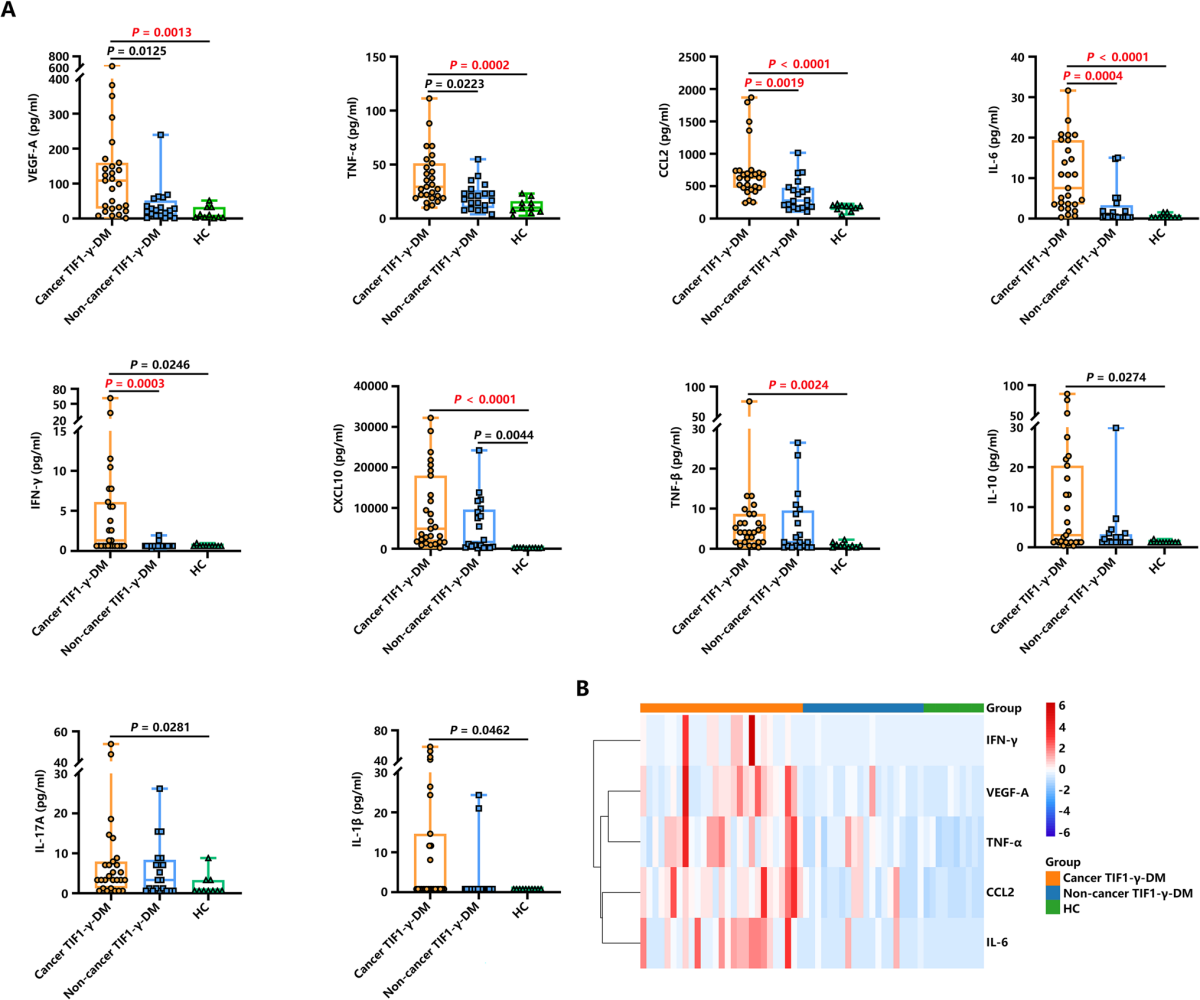
Comparison of the plasma cytokine levels among the Cancer TIF1-γ-DM, Non-cancer TIF1-γ-DM, and HC groups. (**A**) Statistically significant differences within the cytokine levels among the Cancer TIF1-γ-DM (*n* = 27), Non-cancer TIF1-γ-DM (*n* = 20) and HC (*n* = 10) groups. All the data are displayed in boxplots representing the median with the interquartile range. *P* values were determined through the Kruskal-Wallis test followed by Dunn’s multiple comparisons test. *P* < 0.05 indicated statistical significance. *P* < 0.0029 indicated statistical significance after performing Bonferroni correction in bold red text. (**B**) The heatmap of hierarchical cluster analysis is based on the five cytokines among the Cancer TIF1-γ-DM, Non-cancer TIF1-γ-DM, and HC groups.

The plasma levels of 17 inflammatory cytokines were examined (Figure 2). The differences between the Cancer TIF1-γ-DM and the Non-cancer TIF1-γ-DM groups were less pronounced than in the HC group. Notably, VEGF-A, TNF-α, CCL2, IL-6, and IFN-γ were significantly elevated in the Cancer TIF1-γ-DM group than in the Non-cancer TIF1-γ-DM group. CCL2, IL-6, and IFN-γ were also significantly elevated after Bonferroni correction. No differences were observed among other inflammatory cytokines, such as sCD40L, IFN-α2, IL-4, IL-5, CCL3, IL-2, and IL-12p70 among the three groups (Figure S1).

### Upregulated levels of plasma VEGF-A, TNF-α, CCL2, IL-6, and IFN-γ within Untreated Cancer TIF1-γ-DM

**Fig. 3:**
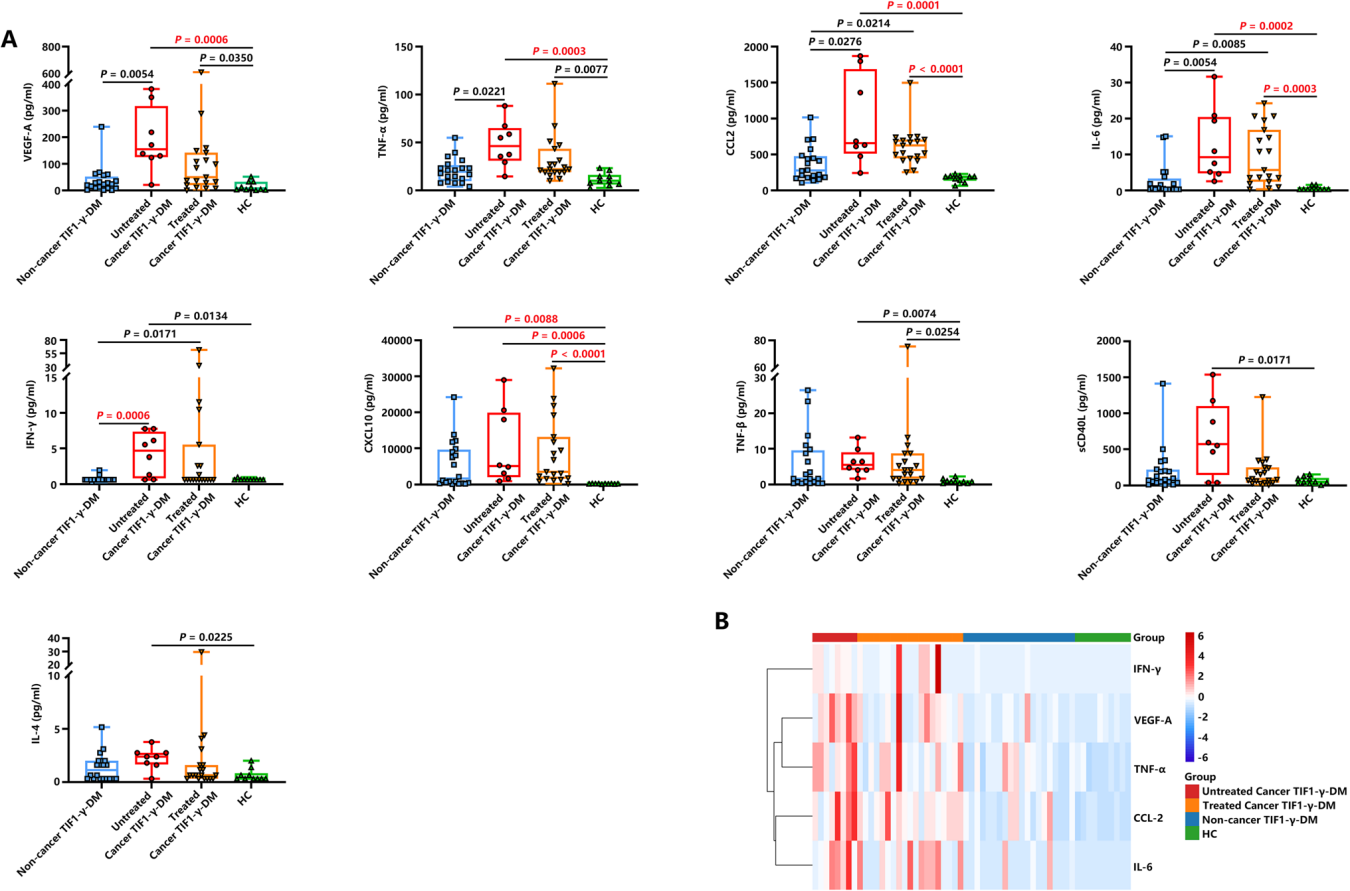
Comparison of the plasma cytokine levels among the four groups. (**A**) Statistically significant differences in the plasma cytokine levels among the Non-cancer TIF1-γ-DM (*n* = 20), Untreated Cancer TIF1-γ-DM (*n* = 8), Treated Cancer TIF1-γ-DM (*n* = 19) and the HC (*n* = 10) groups. All the data are displayed in boxplots representing the median with the interquartile range. *P* values were determined using the Kruskal-Wallis test followed by Dunn’s multiple comparisons test. *P* < 0.05 indicated statistical significance. *P* < 0.0029 indicated statistical significance after performing Bonferroni correction in bold red text. (**B**) The heatmap of hierarchical cluster analysis based on the five cytokines among the Non-cancer TIF1-γ-DM, Untreated Cancer TIF1-γ-DM, Treated Cancer TIF1-γ-DM, and HC groups.

We compared the plasma levels of 17 inflammatory cytokines among the Non-cancer TIF1-γ-DM, Untreated Cancer TIF1-γ-DM, Treated Cancer TIF1-γ-DM, and HC groups (Figure 3) to understand the influence of anticancer treatment on the cytokine levels. Interestingly, plasma VEGF-A, TNF-α, CCL2, IL-6, and IFN-γ cytokines levels were significantly enhanced in the Untreated Cancer TIF1-γ-DM group than in the Non-cancer TIF1-γ-DM group. Additionally, plasma IFN-γ levels were also significantly increased after Bonferroni correction. Plasma CCL2, IL-6, and IFN-γ levels were significantly higher in the Treated Cancer TIF1-γ-DM group than in the Non-cancer TIF1-γ-DM group. Moreover, the cytokine levels were lower in the Treated than in the Untreated Cancer TIF1-γ-DM group, though without any statistical significance. Other plasma cytokine levels, such as IL-10, IL-17A, IL-1β, IFN-α2, IL-5, CCL3, IL-2, and IL-12p70, did not differ significantly among the four groups (Figure S2).

### Distinguish the presence of cancer using circulating VEGF-A, TNF-α, CCL2, IL-6, and IFN-γ in anti-TIF1-γ antibody-positive DM

**Fig. 4:**
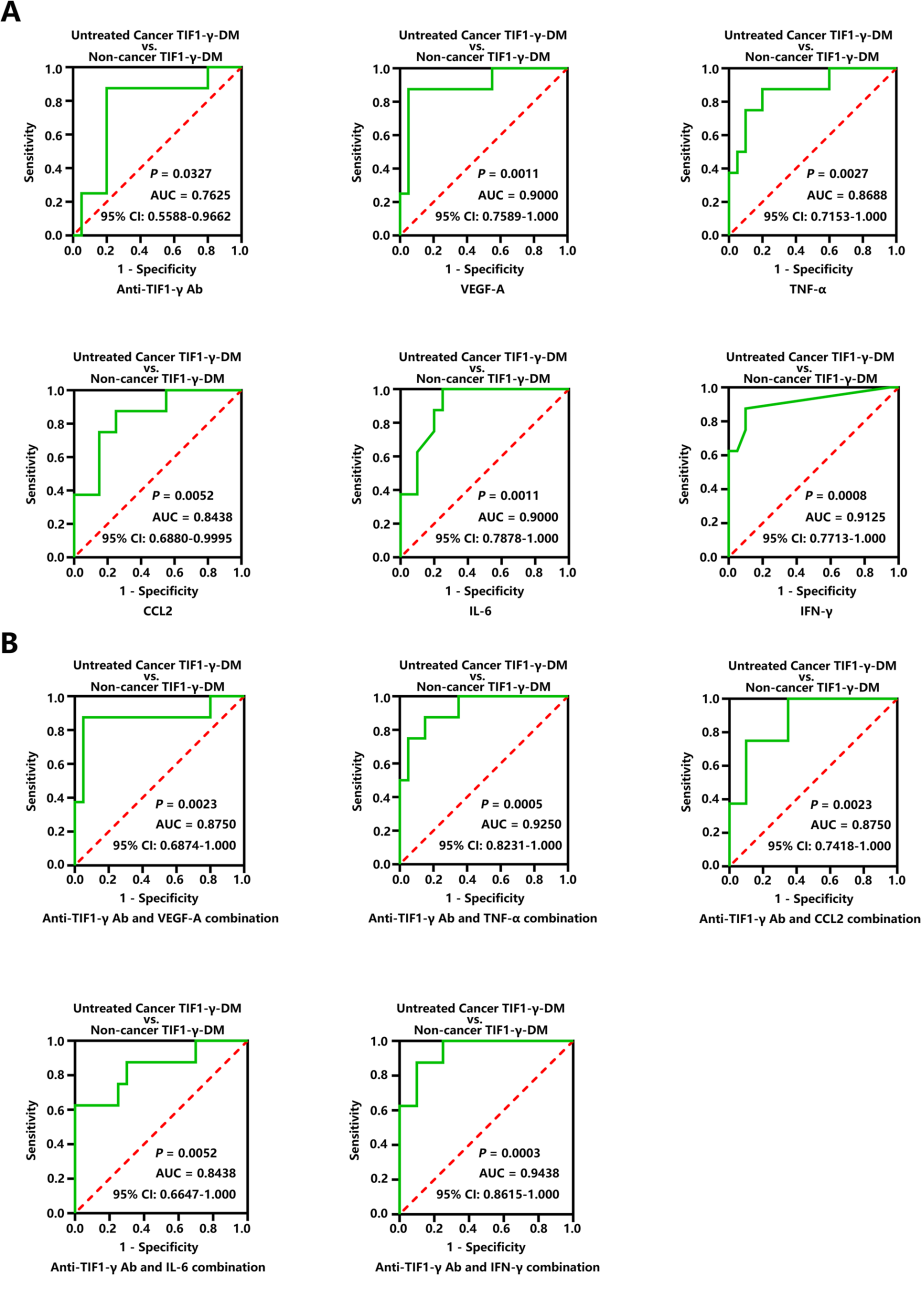
Cytokines reflect the presence of cancer within the anti-TIF1-γ antibody-positive DM. (**A**) ROC curves of the anti-TIF1-γ antibody, VEGF-A, TNF-α and CCL2, IL-6, and IFN-γ for distinguishing the anti-TIF1-γ antibody-positive DM patients having cancer from those without cancer. (**B**) ROC curves of the anti-TIF1-γ antibody combination with VEGF-A, TNF-α, CCL2, IL-6, and IFN-γ, for differentiating the anti-TIF1-γ antibody-positive DM patients having cancer from those without cancer

The higher levels of plasma VEGF-A, TNF-α, CCL2, IL-6, and IFN-γ were depicted in the Untreated Cancer TIF1-γ-DM group than in the Non-cancer TIF1-γ-DM group (Figure 3). Therefore, we hypothesized that VEGF-A, TNF-α, CCL2, IL-6, and IFN-γ could distinguish cancer among anti-TIF1-γ antibody-positive DM patients. The anti-TIF1-γ antibody strongly correlates with an elevated cancer risk among adult DM patients [2]. In addition, ROC curves analysis (Figure 4A) further revealed that plasma anti-TIF1-γ antibody levels with a mild value could distinguish the presence of cancer. The cut-off value of 92.63 U/ml depended on the maximum value of the Youden index, having a sensitivity of 87.5% and a specificity of 80.0% among anti-TIF1-γ antibody-positive DM patients. We determined the cut-off values, sensitivities, and specificities of VEGF-A, TNF-α, CCL2, IL-6, and IFN-γ as biomarkers to investigate whether their plasma levels could distinguish cancer in anti-TIF1-γ antibody-positive DM patients and reflect the presence of cancer through the ROC curves. ROC curves analysis depicted that VEGF-A (Youden index-based cut off 95.77 pg/mL, sensitivity 87.5%, and specificity 95%), TNF-α (Youden index-based cut off 28.37 pg/mL, sensitivity 87.5%, and specificity 80.0%), CCL2 (Youden index-based cut off 462.3 pg/mL, sensitivity 87.5%, and specificity 75.0%), IL-6 (Youden index-based cut off 2.195 pg/mL, sensitivity 100%, and specificity 75%) and IFN-γ (Youden index-based cut off 0.6410 pg/mL, sensitivity 87.5%, and specificity 90%) illuminated an excellent value in identifying cancer among the anti-TIF1-γ antibody-positive DM patients. Besides, the ROC curve analyses further revealed that a combination of anti-TIF1-γ antibody and one of these five cytokines (Figure 4B) also depicted an excellent significance in reflecting the presence of cancer among anti-TIF1-γ antibody-positive DM patients. Nonetheless, the sensitivity, specificity, positive predictive value, and negative predictive value of PET/CT or usual screening are still higher than that of these biomarkers in the Cancer TIF1-γ-DM and Non-cancer TIF1-γ-DM diagnosis (Table S1).

### Positive correlation between circulating cytokines and muscle-associated enzymes in Cancer TIF1-γ-DM

**Fig. 5:**
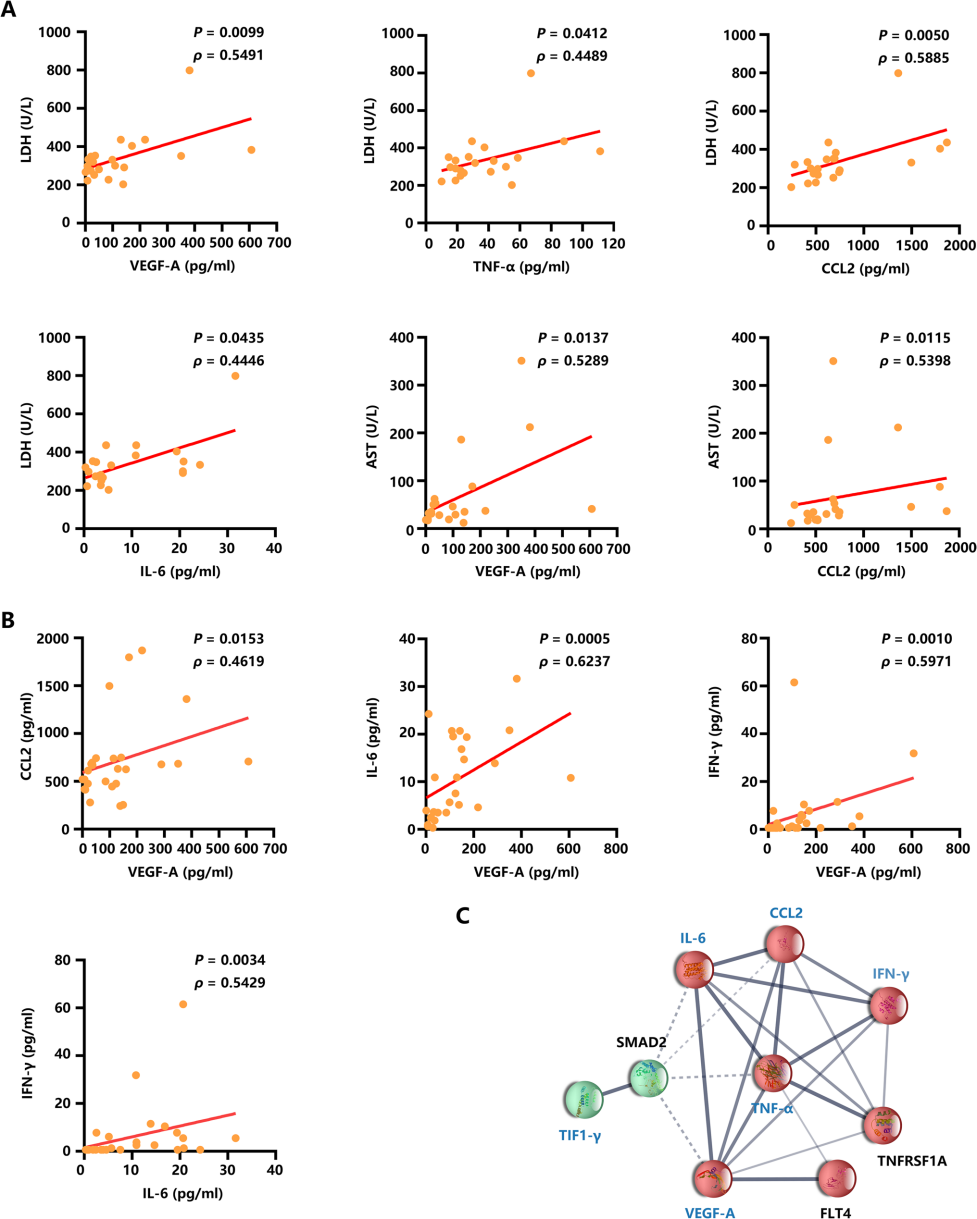
Correlation analyses of cytokine levels and muscle-associated enzymes. (**A**) A statistically significant correlation between LDH and AST levels with cytokine levels among the Cancer TIF1-γ-DM patients. (**B**) Statistically significant correlation among the levels of VEGF-A, TNF-α, CCL2, IL-6, and IFN-γ in Cancer TIF1-γ-DM patients. (**C**) TIF1-γ, VEGF-A, TNF-α, CCL2, IL-6, and IFN-γ protein-protein interaction network based on the STRING database. *P* values were determined using Spearman’s rank correlation test and assessed through Spearman’s correlation coefficient *ρ*. *P* < 0.05 indicated statistical significance

Muscle-associated enzymes LDH and AST were significantly increased in the Cancer TIF1-γ-DM group than in the HC group (Table 1). Then, we analyzed the association of the five cytokines with LDH and AST in Cancer TIF1-γ-DM patients. A significant positive correlation was observed between the LDH levels and the inflammatory cytokines VEGF-A, TNF-α, CCL2, and IL-6 levels within the Cancer TIF1-γ-DM group (Figure 5A). In contrast, there were no correlations between the levels of LDH and IFN-γ within the Cancer TIF1-γ-DM group (Figure S3A). In addition, the AST levels were significantly positively correlated with VEGF-A and CCL2 levels. In contrast, no correlations were observed between the levels of AST and the other three cytokines, TNF-α, IL-6, and IFN-γ, within the Cancer TIF1-γ-DM group (Figure S3A).

Therefore, anti-TIF1-γ antibodies, VEGF-A, TNF-α, CCL2, IL6, and IFN-γ could have essential roles in indicating the presence of cancer in anti-TIF1-γ antibody-positive DM patients (Figure 4A). We investigated and observed a positive correlation between VEGF-A and CCL2 levels, VEGF-A and IL-6 levels, VEGF-A and IFN-γ levels, and IL-6 and IFN-γ levels within the Cancer TIF1-γ-DM group (Figure 5B). A statistically significant correlation was not observed among other cytokines (Figure S3B). Additionally, we observed a statistically insignificant correlation between anti-TIF1-γ antibody levels and these five cytokines (Figure S3C). Interestingly, TIF1-γ, VEGF-A, TNF-α, CCL2, IL-6, and IFN-γ proteins could have some interactions through the STRING 11.5 database (Figure 5C).

## Discussion

Although anti-TIF1-γ antibody-positive adult DM patients are at elevated cancer risk, not all anti-TIF1-γ antibody-positive DM patients develop cancer. Tumor markers, such as CEA, CA15-3, and CA19-9, in addition to CA125, could not help in CAM. Therefore, discovering biomarkers could help detect and screen for occult cancer. In this study, high levels of plasma VEGF-A, TNF-α, CCL2, IL-6, and IFN-γ in the Cancer TIF1-γ-DM group (Figure 2), particularly those not undergoing any anticancer treatment (Figure 3), were excellent in distinguishing cancer among the anti-TIF1-γ antibody-positive DM patients (Figure 4A). Anti-TIF1-γ antibody and IFN-γ had the best combination in differentiating the Untreated Cancer TIF1-γ-DM group from the Non-cancer DM one (Figure 4B). Furthermore, the STRING network formed a connected protein-protein interaction between TIF1-γ and the five cytokines (Figure 5C), indicating their involvement in the Cancer TIF1-γ-DM process through interaction.

Herein, higher levels of ESR were observed in the Cancer TIF1-γ-DM group than in the Non-cancer TIF1-γ-DM group. It has been reported that elevated ESR is correlated with cancer-associated DM [25]. Although there was a higher proportion of males in the Cancer TIF1-γ-DM group than in the Non-cancer TIF1-γ-DM group in our study, ESR is typically more elevated in females than in males [26]. It further confirmed that the enhancement of ESR is associated with Cancer TIF1-γ-DM. Muscle-associated enzymes LDH and AST, but not CK and ALT, were found elevated and positively correlated with some cytokines in the Cancer TIF1-γ-DM group (Table 1 and Figure 5). Thus, LDH and AST were not only involved in muscle inflammation among the Cancer TIF1-γ-DM patients but also were potentially reflective of underlying cancer. This potential relation remains to be explored in the future. Several studies indicated that serum levels of anti-TIF1-γ antibody reduced after successful cancer treatment and elevated with cancer worsening in DM patients [22, 27, 28]. In a study by IKEDA et al., the serum levels of anti-TIF1-γ antibody did not depict a significant difference between the patients with and without cancer [22]. However, Ly et al. observed that serum levels of anti-TIF1-γ antibodies were significantly higher in the Cancer TIF1-γ-DM group than in the Non-cancer DM group [16]. The results were consistent with our findings (Figure 1A), indicating that anti-TIF1-γ antibody levels could reflect cancer risk. In contrast, no significant differences were observed in the anti-TIF1-γ antibody levels among the Untreated and the Treated Cancer TIF1-γ-DM group.

Although the hypothesis that the cross-reaction of anti-tumor response could cause myositis and that not all myositis patients develop cancer due to the cancer immunoediting theory are well known [29], the detailed pathogenic CAM mechanisms remain obscure. VEGF-A is also referred to as VEGF and is produced by multiple cells such as M2-like macrophages and tumor cells, and is as a critical driver of the angiogenic switch. It has been observed to be elevated in the plasma of patients with various types of cancer [30]. Previous studies have unveiled that VEGF levels were more significantly elevated in serum and muscle tissues of DM patients than in healthy controls, depicting the involvement of angiogenic cytokine VEGF during the angiogenic process of DM [31, 32]. Although the plasma VEGF-A levels were not significantly different between the anti-TIF1-γ antibody-positive DM patients without cancer and the healthy controls, our data depicted that VEGF-A levels were significantly upregulated in the Cancer TIF1-γ-DM group than in the Non-cancer TIF1-γ-DM and HC groups (Figures 2 and 3). It indicated that cancer could lead to elevated VEGF-A levels. Moreover, plasma VEGF-A levels higher than 95.77 pg/mL could reflect the presence of cancer in anti-TIF1-γ antibody-positive DM patients, depicting that quantifying VEGF-A in plasma could facilitate patient identification of cancer risk among anti-TIF1-γ antibody-positive DM patients.

TNF-α (also known as TNF), a major pro-inflammatory cytokine primarily produced by activated macrophages, T-lymphocytes, and tumor cells, was involved in the pathological processes of DM and cancer. High plasma TNF levels have been observed in some cancer patients with poor prognoses [33]. TNF-α mRNA-positive infiltrating cells more frequently occurred in DM muscles. It is suggested that TNF-α could participate in the pathogenic DM process [34]. Elevated TNF-α serum levels have been shown in anti-TIF1-γ antibody-positive DM patients suffering from significant muscle weakness and elevated cancer rate [35]. Our results were consistent with this finding which further revealed that plasma TNF-α levels in the Cancer TIF1-γ-DM group were significantly higher than in the Non-cancer TIF1-γ-DM and HC groups (Figures 2 and 3). Moreover, TNF-α could associate inflammation with cancer in DM. Plasma TNF-α levels higher than 28.37 pg/mL could identify cancer in anti-TIF1-γ antibody-positive DM patients (Figure 4A). In addition, the anti-TIF1-γ antibody and TNF-α combination depicted a high AUC for cancer existence in anti-TIF1-γ antibody-positive DM patients (Figure 4B).

CCL2, as a pro-inflammatory cytokine, is secreted by the tumor, endothelial, fibroblasts, epithelial, smooth muscle, mesangial, astrocytic, monocytic, and microglial cells and participates in the pathogenesis of various cancers and autoimmune diseases. Endothelial CCL2 protein, not CCL2 mRNA, is reported to be strongly expressed by perifascicular and perimysial endothelia of DM [36]. Some cancer patients have frequently upregulated serum and plasma CCL2 levels [37, 38]. Previous studies revealed that serum CCL2 levels were significantly higher among DM and anti-TIF1-γ antibody-positive DM patients than in healthy controls. Consistent with these findings, we further observed that plasma CCL2 levels were significantly higher within the Cancer TIF1-γ-DM group than in the Non-cancer TIF1-γ-DM and HC groups (Figures 2 and 3). In addition, CCL2 could contribute to cancer development in anti-TIF1-γ antibody-positive DM patients. Plasma levels of CCL2 > 462.3 pg/mL were considered suitable to reflect cancer risk in anti-TIF1-γ antibody-positive DM patients (Figure 4A).

Elevated serum IL-6 levels are associated with DM disease activity, and enhanced serum IFN-γ levels have been associated with LDH in anti-MDA5 antibody-positive DM [39]. Moreover, dysregulated IL-6 expression is frequently observed in cancer [40]. The effects of IFN-γ on anti- and pro-tumor have also been described [41]. Our study found that plasma IL-6 and IFN-γ levels were significantly higher within the Cancer TIF1-γ-DM group than in the Non-cancer TIF1-γ-DM and HC groups (Figures 2 and 3). In addition, plasma levels of IL-6 and IFN-γ could differentiate cancer presence among anti-TIF1-γ antibody-positive DM patients (Figure 4A).

Tumor-associated macrophages (TAMs) inside the tumor microenvironment (TME) are critical as essential mediators for correlating inflammation with cancer. Human cancer cells produce CCL2 and TNF-α. CCR2 ^High^ monocytes guided through the CCR2-CCL2 axis within the tumors facilitate cancer cell survival through VEGF production [42]. TAMs promote angiogenesis by producing angiogenic factor VEGF-A and regulating cancer cell metastasis [43]. TNF-α could also increase the expression of VEGF-A [44]. IL-6, a pro-tumorigenic cytokine, has been suggested to be involved in cancer initiation and rapid progression through potentiating VEGF [40]. Interestingly, TIF1-γ potentially interacted with VEGF-A, TNF-α, CCL2, IL-6, and IFN-γ based on the results of the protein-protein interaction network (Figure 5C). Furthermore, significant positive correlations were observed among VEGF-A and three cytokines, such as CCL2, IL-6, and IFN-γ, and a positive association between IL-6 and IFN-γ within the Cancer TIF1-γ-DM group (Figure 5B). The upregulation of plasma levels of VEGF-A, TNF-α, CCL2, IL-6, and IFN-γ in Cancer TIF1-γ-DM patients could provide insight into the underlying mechanism of the pathogenesis of Cancer TIF1-γ-DM. TIF1-γ mutation or overexpression in cancer cells was hypothesized activate CCL2 and TNF-α expression. Moreover, TIF1-γ-DM chronic exposure to IL-6 and IFN-γ could further the initiation and progression of cancer. In addition, TIF1-γ-induced CCL2 upregulation through the CCR2-CCL2 axis recruited macrophages into the tumor, promoting angiogenesis through VEGF-A excretion (Graphical abstract). Additional studies are required to clarify the correlation between TIF1-γ mutation or overexpression and these five cytokines inside the tumor tissues of Cancer TIF1-γ-DM patients. Therefore, the detailed pathogenic molecular mechanism of Cancer TIF1-γ-DM requires further investigation.

However, our study had several limitations. Since it is a retrospective observational study, it is not allowed to state that these biomarkers are predictors of cancer development. This work is largely exploratory, and a prospective large cohort study is necessary to validate the value of biomarkers in future Cancer TIF1-γ-DM patients. Age and sex significantly differed between the Cancer TIF1-γ-DM and the Non-cancer TIF1-γ-DM groups. A higher risk of cancer in IIM of older age and male sex could be the reason for the discrepancy in distribution [45]. We did not perform the logistic regression to adjust the sex and age confounders in this study due to the limited small sizes insufficient for effective analysis. Our major results were consistent with the significant results stratified with only women (Figure S4) or only patients ≥ 50 years old at DM diagnosis (Figure S5). Cancer TIF1-γ-DM is a rare disease. Therefore, plasma samples within the Untreated and Treated Cancer TIF1-γ-DM groups for cancer treatment were not obtained from the same patients. Furthermore, 46 out of 47 patients had undergone DM treatment before plasma collection. Cytokine levels could have fluctuated after treatment, although no significant difference was observed in the medication differences regarding DM between the Cancer TIF1-γ-DM and the Non-cancer TIF1-γ-DM groups (Table 1), cancer treatment may influence cytokine levels. Of particular concern is that CCL2, IL-6, and IFN-γ were still significantly increased after Bonferroni correction in the Cancer TIF1-γ-DM group. These cytokines are more likely to be biologically relevant.

## Conclusion

The current study identified VEGF-A, TNF-α, CCL2, IL-6, and IFN-γ (CCL2, IL-6, and IFN-γ after Bonferroni correction) as additional plasma biomarkers reflecting cancer presence, in addition to anti-TIF1-γ antibody, among anti-TIF1-γ antibody-positive DM patients. Therefore, these findings indicate a more detailed cytokine profile during diagnosis and provide additional support for cancer screening strategies and potential therapeutic targets for clinical treatment of Cancer TIF1-γ-DM. Moreover, our findings provide a novel perspective on Cancer TIF1-γ-DM pathogenesis.

## Supplementary information

Fig. S1 Comparison of the plasma cytokine levels among the Cancer TIF1-γ-DM, Non-cancer TIF1-γ-DM, and HC groups. Statistically insignificant differences in the cytokine levels among the Cancer TIF1-γ-DM (n = 27), Non-cancer TIF1-γ-DM (n = 20) and HC (n = 10) groups. All the data are displayed in boxplots representing the median with the interquartile range. *P* values were obtained using the Kruskal-Wallis test followed by Dunn’s multiple comparisons test. *P* < 0.05 indicated statistical significance. *P* < 0.0029 indicated statistical significance after performing Bonferroni correction in bold red text. (PNG 218 kb)

High Resolution Image (TIF 1518 kb)

Fig. S2 Comparison of the plasma cytokine levels among the four groups. Statistically insignificant differences in the cytokine levels among the Non-cancer TIF1-γ-DM (*n* = 20), Untreated Cancer TIF1-γ-DM (*n* = 8), Treated Cancer TIF1-γ-DM (*n* =19) and HC (*n* = 10) groups. All the data are displayed in boxplots representing the median with the interquartile range. *P* values were determined using the Kruskal-Wallis test followed by Dunn’s multiple comparisons test. *P* < 0.05 indicated statistical significance. *P* < 0.0029 indicated statistical significance after performing Bonferroni correction in bold red text. (PNG 242 kb)

High Resolution Image (TIF 1621 kb)

Fig. S3 Correlation analyses of cytokine levels and muscle-associated enzymes. (A) Statistically insignificant correlation between the LDH and AST levels with cytokine levels in the Cancer TIF1-γ-DM patients. (B) Statistically insignificant correlation among the levels of VEGF-A, TNF-α, CCL2, IL-6, and IFN-γ in the Cancer TIF1-γ-DM patients. (C) Statistically insignificant correlation between the levels of anti-TIF1-γ antibody with the levels of VEGF-A, TNF-α, CCL2, IL-6, and IFN-γ in the Cancer TIF1-γ-DM patients. *P* values were determined using Spearman’s rank correlation test and assessed using Spearman’s correlation coefficient *ρ*. *P* < 0.05 indicated statistical significance. (PNG 335 kb)

High Resolution Image (TIF 1582 kb)

Fig. S4 Comparison of the plasma cytokine levels between the Cancer TIF1-γ-DM (*n* = 17) and the Non-cancer TIF1-γ-DM groups (*n* = 20) stratified with only women. *P* values were evaluated through the Mann-Whitney *U* test. All the data are displayed in boxplots depicting the median with an interquartile range. *P* < 0.05 indicated statistical significance. *P* < 0.0029 indicated statistical significance after performing Bonferroni correction in bold red text. (PNG 433 kb)

High Resolution Image (TIF 2281 kb)

Fig. S5 Comparison of the plasma cytokine levels between the Cancer TIF1-γ-DM (*n* = 22) and the Non-cancer TIF1-γ-DM groups (*n* = 10) stratified only with patients ≥ 50 years old at DM diagnosis. *P* values were calculated using the Mann-Whitney *U* test. All the data are displayed in boxplots representing the median with an interquartile range. *P* < 0.05 indicated statistical significance. *P* < 0.0029 indicated statistical significance after performing Bonferroni correction in bold red text. (PNG 435 kb)

High Resolution Image (TIF 2354 kb)

ESM 1 (DOCX 17.1 kb)
