# Supplementary figures and images for: Circulating VEGF-A, TNF-α, CCL2, IL-6, and IFN-γ as biomarkers of cancer in cancer-associated anti-TIF1-γ antibody-positive dermatomyositis
Source: Clin Rheumatol. 2022 Nov 11;42(3):817–30. doi: 10.1007/s10067-022-06425-3 (PMC9935732; doi:10.1007/s10067-022-06425-3)

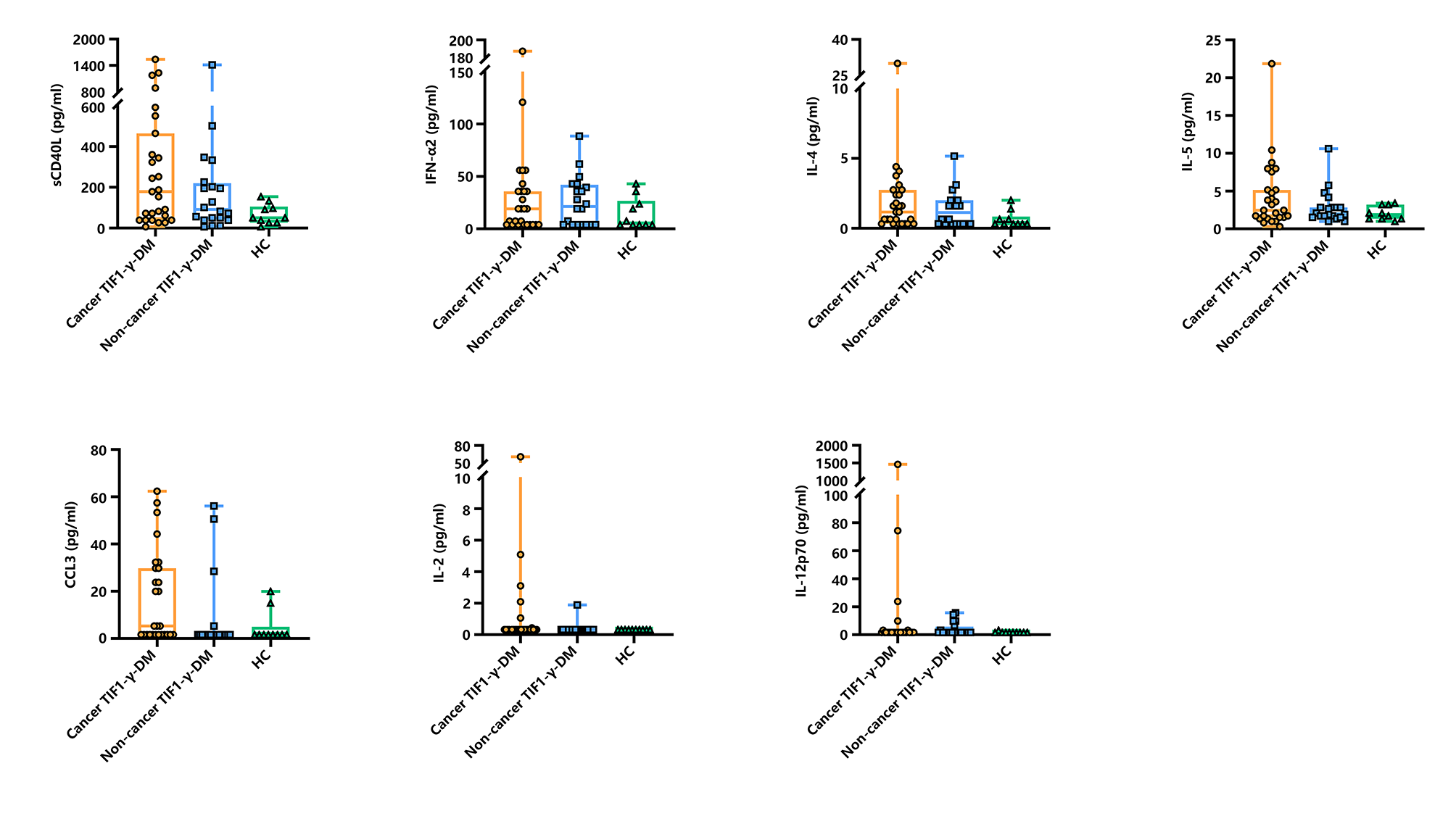

Supplement: Supplementary file 1 — Comparison of the plasma cytokine levels among the Cancer TIF1-γ-DM, Non-cancer TIF1-γ-DM, and HC groups. Statistically insignificant differences in the cytokine levels among the Cancer TIF1-γ-DM (n = 27), Non-cancer TIF1-γ-DM (n = 20) and HC (n = 10) groups. All the data are displayed in boxplots representing the median with the interquartile range. P values were obtained using the Kruskal-Wallis test followed by Dunn’s multiple comparisons test. P < 0.05 indicated statistical significance. P < 0.0029 indicated statistical significance after performing Bonferroni correction in bold red text. (PNG 218 kb) [file 10067_2022_6425_Fig6_ESM.png]

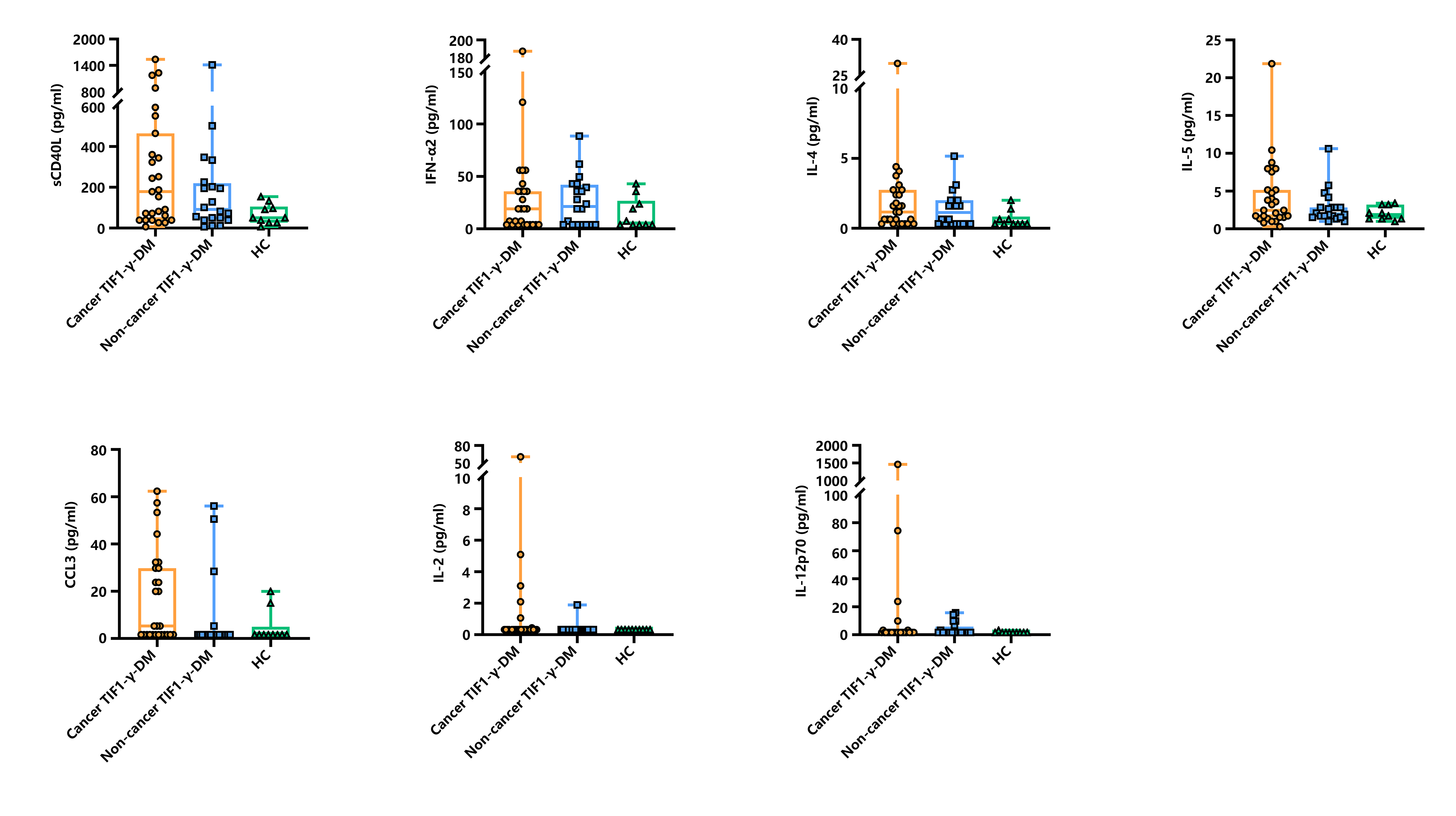

Supplement: Supplementary file 2 — High Resolution Image (TIF 1518 kb) [file 10067_2022_6425_MOESM1_ESM.tif]

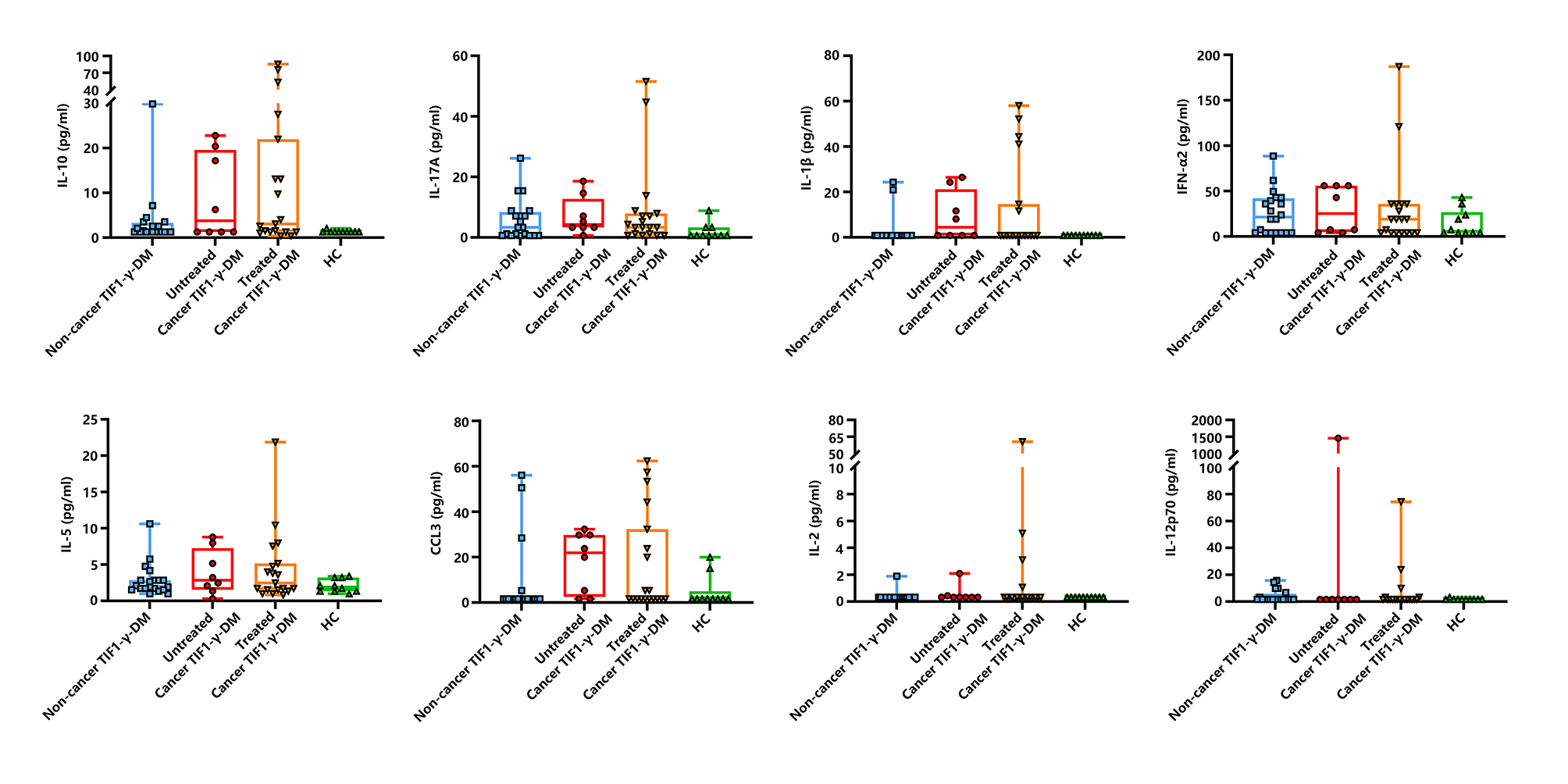

Supplement: Supplementary file 3 — Comparison of the plasma cytokine levels among the four groups. Statistically insignificant differences in the cytokine levels among the Non-cancer TIF1-γ-DM (n = 20), Untreated Cancer TIF1-γ-DM (n = 8), Treated Cancer TIF1-γ-DM (n =19) and HC (n = 10) groups. All the data are displayed in boxplots representing the median with the interquartile range. P values were determined using the Kruskal-Wallis test followed by Dunn’s multiple comparisons test. P < 0.05 indicated statistical significance. P < 0.0029 indicated statistical significance after performing Bonferroni correction in bold red text. (PNG 242 kb) [file 10067_2022_6425_Fig7_ESM.png]

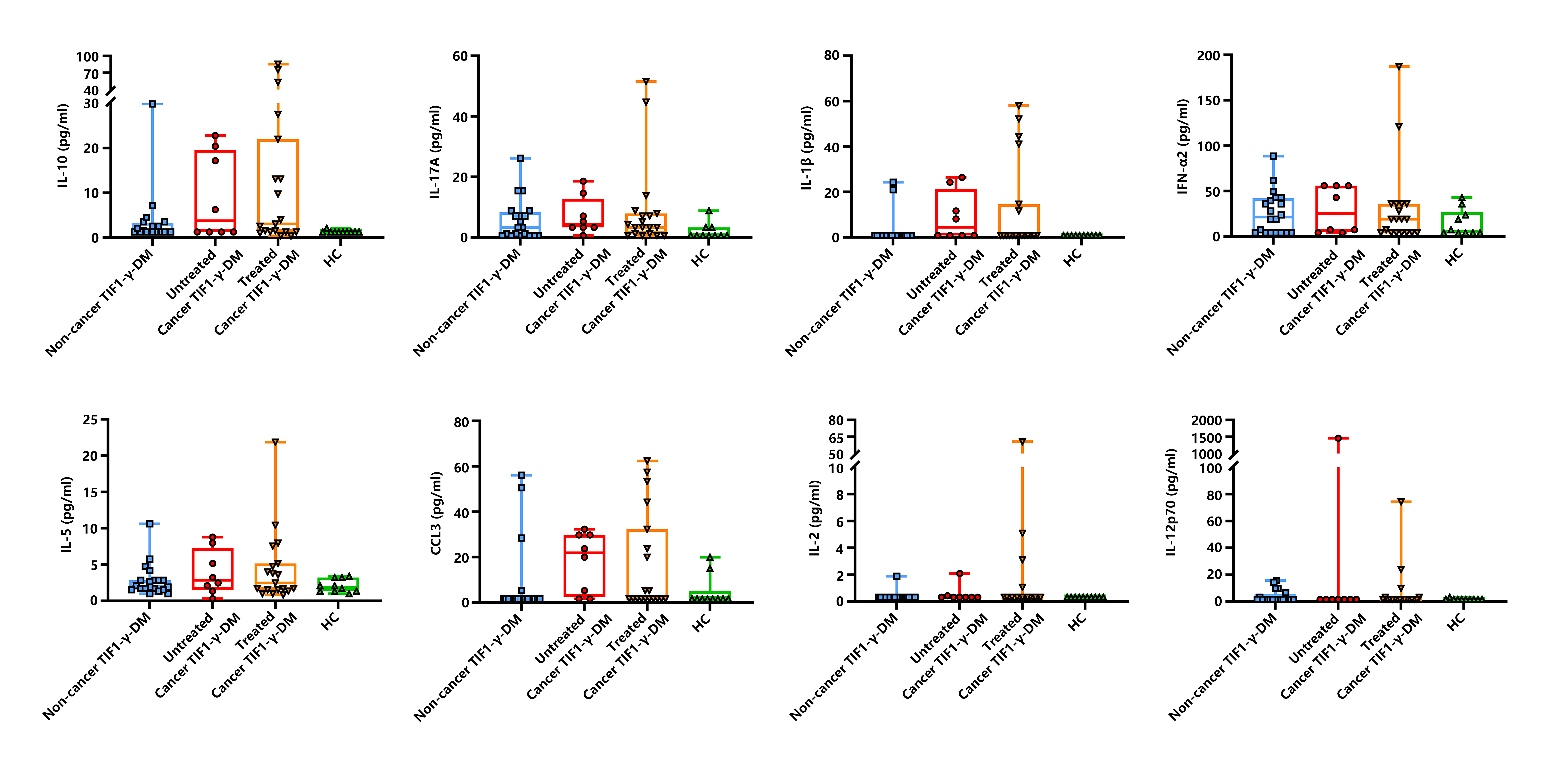

Supplement: Supplementary file 4 — High Resolution Image (TIF 1621 kb) [file 10067_2022_6425_MOESM2_ESM.tif]

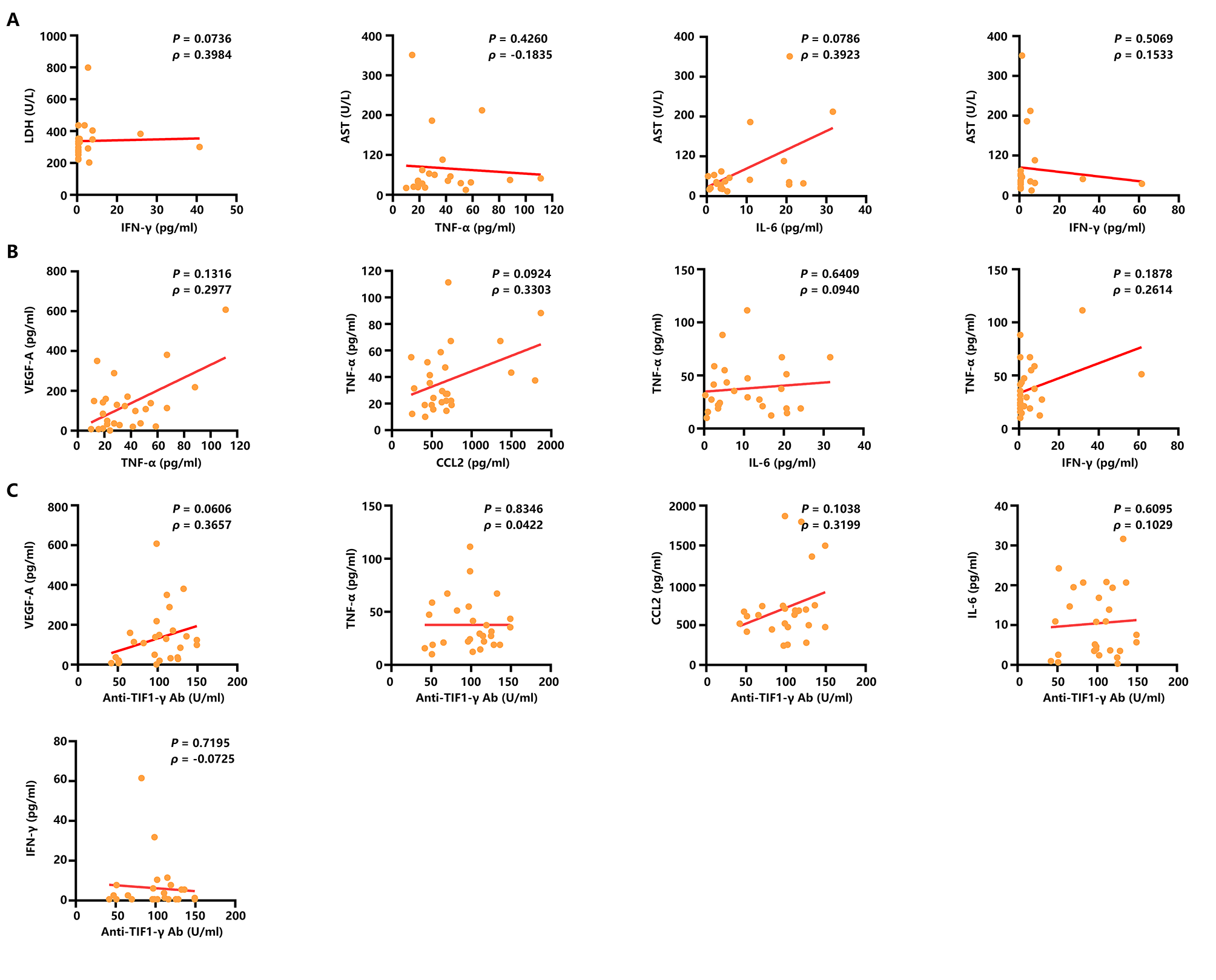

Supplement: Supplementary file 5 — Correlation analyses of cytokine levels and muscle-associated enzymes. (A) Statistically insignificant correlation between the LDH and AST levels with cytokine levels in the Cancer TIF1-γ-DM patients. (B) Statistically insignificant correlation among the levels of VEGF-A, TNF-α, CCL2, IL-6, and IFN-γ in the Cancer TIF1-γ-DM patients. (C) Statistically insignificant correlation between the levels of anti-TIF1-γ antibody with the levels of VEGF-A, TNF-α, CCL2, IL-6, and IFN-γ in the Cancer TIF1-γ-DM patients. P values were determined using Spearman’s rank correlation test and assessed using Spearman’s correlation coefficient ρ. P < 0.05 indicated statistical significance. (PNG 335 kb) [file 10067_2022_6425_Fig8_ESM.png]

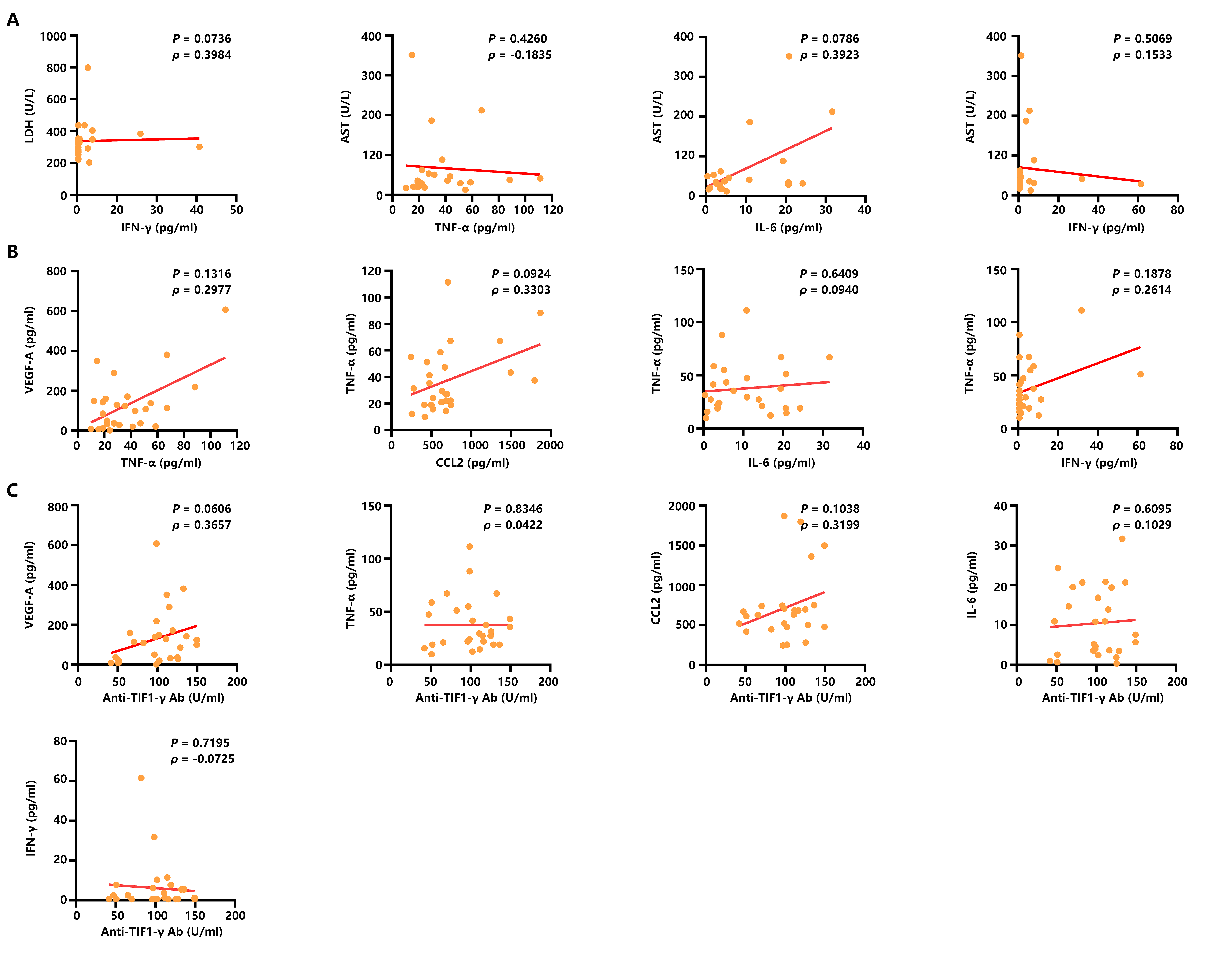

Supplement: Supplementary file 6 — High Resolution Image (TIF 1582 kb) [file 10067_2022_6425_MOESM3_ESM.tif]

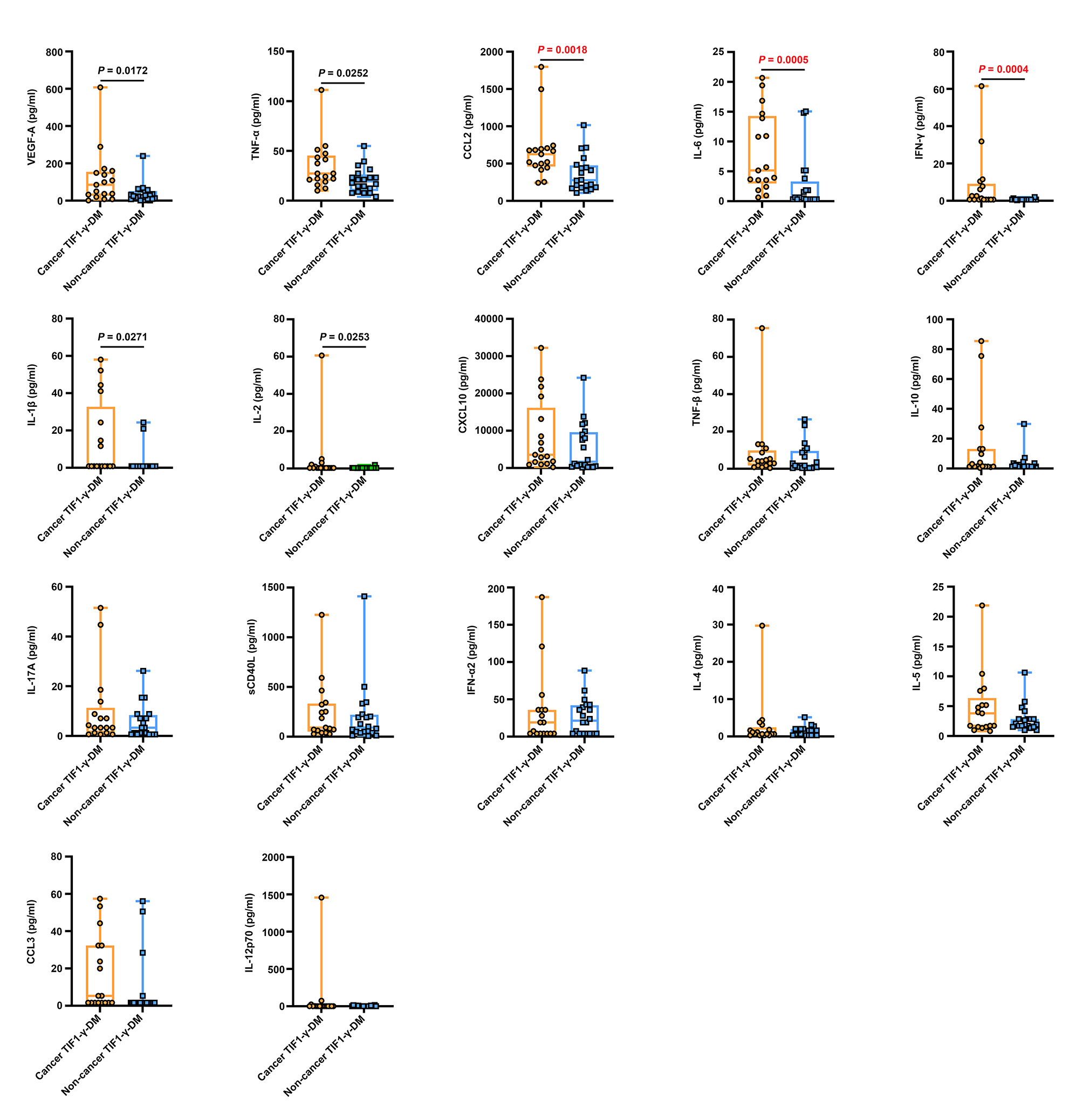

Supplement: Supplementary file 7 — Comparison of the plasma cytokine levels between the Cancer TIF1-γ-DM (n = 17) and the Non-cancer TIF1-γ-DM groups (n = 20) stratified with only women. P values were evaluated through the Mann-Whitney U test. All the data are displayed in boxplots depicting the median with an interquartile range. P < 0.05 indicated statistical significance. P < 0.0029 indicated statistical significance after performing Bonferroni correction in bold red text. (PNG 433 kb) [file 10067_2022_6425_Fig9_ESM.png]

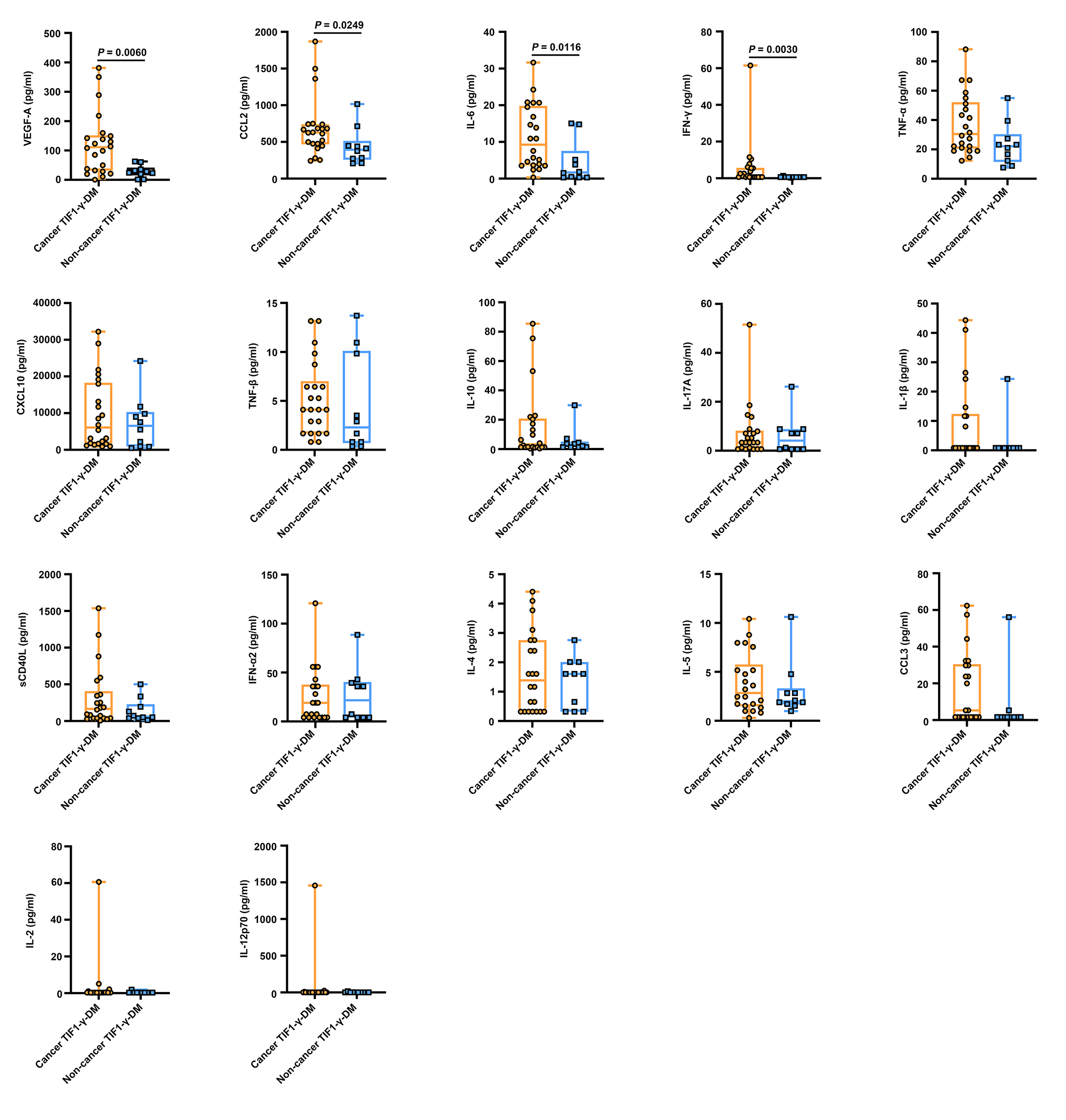

Supplement: Supplementary file 9 — Comparison of the plasma cytokine levels between the Cancer TIF1-γ-DM (n = 22) and the Non-cancer TIF1-γ-DM groups (n = 10) stratified only with patients ≥ 50 years old at DM diagnosis. P values were calculated using the Mann-Whitney U test. All the data are displayed in boxplots representing the median with an interquartile range. P < 0.05 indicated statistical significance. P < 0.0029 indicated statistical significance after performing Bonferroni correction in bold red text. (PNG 435 kb) [file 10067_2022_6425_Fig10_ESM.png]
