# Supplementary material for: Circulating VEGF-A, TNF-α, CCL2, IL-6, and IFN-γ as biomarkers of cancer in cancer-associated anti-TIF1-γ antibody-positive dermatomyositis
Source: Clin Rheumatol. 2022 Nov 11;42(3):817–30. doi: 10.1007/s10067-022-06425-3 (PMC9935732; doi:10.1007/s10067-022-06425-3)
Supplement: Supplementary file 11 — (DOCX 17.1 kb) [file 10067_2022_6425_MOESM6_ESM.docx]

**Table S1. Diagnostic performance of PET/CT or usual screening and biomarkers for Cancer TIF1-γ-DM (Cancer TIF1-γ-DM vs. Non-cancer TIF1-γ-DM)**

| Parameter | Sensitivity (%) | Specificity (%) | PPV (%) | NPV (%) |
| --- | --- | --- | --- | --- |
| PET/CT or usual screening | 86 | 100 | 100 | 91 |
| Anti-TIF1-γ antibody | 70 | 80 | 83 | 67 |
| VEGF-A | 56 | 95 | 94 | 61 |
| TNF-α | 52 | 80 | 78 | 55 |
| CCL2 | 74 | 75 | 80 | 68 |
| IL-6 | 85 | 75 | 82 | 79 |
| IFN-γ | 63 | 85 | 85 | 63 |

*PPV*, positive predictive value, *NPV*, negative predictive value
